# Traction Performance Evaluation of the Electric All-Wheel-Drive Tractor

**DOI:** 10.3390/s22030785

**Published:** 2022-01-20

**Authors:** Seung-Yun Baek, Seung-Min Baek, Hyeon-Ho Jeon, Wan-Soo Kim, Yeon-Soo Kim, Tae-Yong Sim, Kyu-Hong Choi, Soon-Jung Hong, Hyunggun Kim, Yong-Joo Kim

**Affiliations:** 1 Department of Smart Agriculture Systems, Chungnam National University, Daejeon 34134, Korea; kelpie0037@gmail.com (S.-Y.B.); bsm1104@naver.com (S.-M.B.); jhh5888@naver.com (H.-H.J.); 2 Department of Biosystems Machinery Engineering, Chungnam National University, Daejeon 34134, Korea; wskim0726@gmail.com; 3 Convergence Agricultural Machinery Group, Korea Institute of Industrial Technology (KITECH), Gimje 54325, Korea; kimtech612@gmail.com; 4 Department of Artificial Intelligence, Sejong University, Seoul 05006, Korea; tysim@sejong.ac.kr; 5 Department of Smart Agriculture, Jeonju University, Jeonju 55069, Korea; niaechoi@jj.ac.kr; 6 Department of General Education, Korea National College of Agriculture and Fisheries, Jeonju 54874, Korea; hsj43333@korea.kr; 7 Department of Biomechatronic Engineering, Sungkyunkwan University, Suwon 16419, Korea; hkim.bme@skku.edu

**Keywords:** electric AWD tractor, traction performance, traction test, load measurement system

## Abstract

This study aims to design, develop, and evaluate the traction performance of an electric all-wheel-drive (AWD) tractor based on the power transmission and electric systems. The power transmission system includes the electric motor, helical gear reducer, planetary gear reducer, and tires. The electric system consists of a battery pack and charging system. An engine-generator and charger are installed to supply electric energy in emergency situations. The load measurement system consists of analog (current) and digital (battery voltage and rotational speed of the electric motor) components using a controller area network (CAN) bus. A traction test of the electric AWD tractor was performed towing a test vehicle. The output torques of the tractor motors during the traction test were calculated using the current and torque curves provided by the motor manufacturer. The agricultural work performance is verified by comparing the torque and rpm (T–N) curve of the motor with the reduction ratio applied. The traction is calculated using torque and specifications of the wheel, and traction performance is evaluated using tractive efficiency (TE) and dynamic ratio (DR). The results suggest a direction for the improvement of the electric drive system in agricultural research by comparison with the conventional tractor through the analysis of the agricultural performance and traction performance of the electric AWD tractor.

## 1. Introduction

The fuel consumption of agricultural machinery continues to increase in Korea, increasing the seriousness of environmental pollution [1,2]; accordingly, the Technology Innovation and Emissions Reduction (TIER) regulations for Diesel engines have been strengthened. To combat these actions, research into the development of an electric drive power transmission system for agricultural machinery is actively in progress [3,4,5,6]. In particular, related studies are expected to continuously increase [7] based on the results of a study showing that electric vehicles (EVs) reduce CO_2_ emissions by approximately 30~70% compared to the internal combustion engine when considering the entire process of EV production, use, and disposal. An electric vehicle using an electric motor can be classified as hybrid electric vehicle (HEV), such as serial hybrids and parallel hybrids, and battery electric vehicle (BEV) [8,9,10]. The serial and parallel hybrids use an electric motor in common, but the internal combustion engine of the serial hybrid is only used for charging the battery, and the electric motor drives the platform [11]. By contrast, the internal combustion engine and an electric motor of the parallel hybrid type are selectively used by a control method depending on the speed and load of the platform. The BEV type is a method of removing the internal combustion engine for battery charging and using a charging system and regenerative braking for battery charging and driving the vehicle [10,12]. The development of automobiles with HEVs and BEVs has been studied for several years [13,14,15]. However, in the field of agricultural machines, the commercialization of tractors with electric drive power transmission systems is basically nonexistent, and the market share of conventional Diesel engine tractors is still much higher. The application of an electric drive power transmission system to a tractor has limitations for all three types (serial hybrid, parallel hybrid, and battery electric). High-torque driving motor technology is not yet suitable for application in serial hybrid and battery electric tractors. The energy conversion efficiency of an electric motor varies with load in irregular environments such as soil, making it difficult to maintain optimum efficiency [16]. The parallel hybrid type struggles to properly distribute power because the driving motor must develop power and torque to match the load [17]. In addition, BEVs require expensive and large-capacity batteries for functioning [18]. Because of the load occurring during agricultural work, the motor consumes high power, making it difficult to perform continuous work on a single charge. Electric drive technology has been studied for the development of electric all-wheel-drive (AWD) vehicles, in which four motors are used, one for each quadrant of the drive shafts [19,20]. An electric motor with a small torque capacity can easily be applied because the electric AWD tractor uses four motors, one for each axle quadrant, and each electric motor manages the torque on its axle quadrant. More research on AWD tractors using four motors is needed, especially research on traction performance. Traction performance is an important tractor metric for agricultural equipment [21]. It determines the draft force of the attached implement and is crucial to effective tractor design [22]. A tractor is a vehicle that does agricultural work through power take-off (PTO) as well as towing work. However, the electric AWD tractor was designed for transport operations and plowing. Therefore, we decided that traction performance was the most important factor on this platform. Traction performance varies with tractor specifications, drive shaft load distribution, tires, and hitch type [23]. Tractor tests conducted using the Organization for Economic Cooperation and Development (OECD) test code are typically performed on a concrete tractive surface, to attain relatively consistent results. Traction performance has been evaluated on concrete, and an important aspect is the slip of the tires or tracks [24]. Typical indicators for evaluating traction performance are tractive efficiency (TE) and dynamic ratio (DR) [24]. The TE is the ratio of the drawbar power to the axle power, so it is an indicator of the ability of the tractor to transfer power from the axle to the drawbar. The DR is the ratio between drawbar pull and the weight of the tractor [21]. A number of studies have been conducted on the traction performance of four-wheel-drive tractors [25,26], but few studies have been conducted on electric drive AWD tractors equipped with electric motors driving the axles [20]. The electric tractor uses a motor as its main driving source, and similar to a tractor powered by an internal combustion engine, the output torque capacity is limited and the output is determined by the workload [27,28]. Performance evaluation is essential because the power of the motor influences the working performance of an electric tractor. This study aims to provide useful information for the design optimization of the electric AWD tractors by designing and developing an electric AWD tractor based on the power transmission and electric systems. In addition, a load measurement system is developed to measure the load data of the electric AWD tractor. The load data are obtained through traction tests, and the traction performance is analyzed and compared with previous study results based on the output torque of the electric motor, TE, and DR.

## 2. Materials and Methods

### 2.1. Electric AWD Tractor

**Figure 1:**
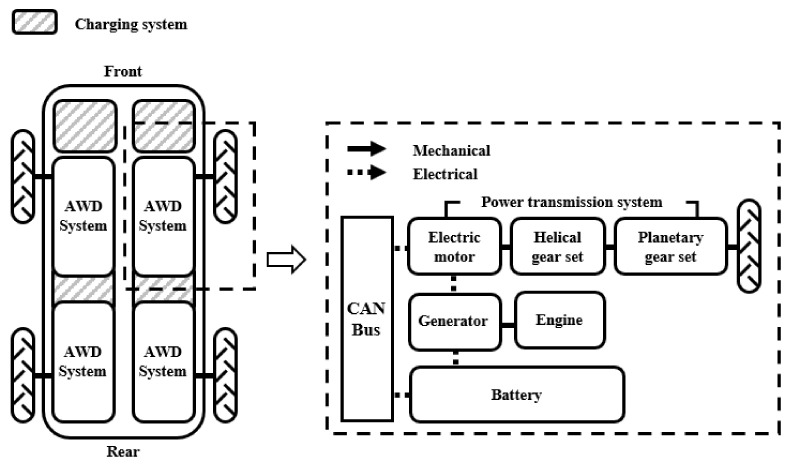
Schematic of the power transmission system of the electric AWD tractor.

The electric motor of the AWD tractor used in this study replaced the conventional Diesel engine, which is the driving source of the agricultural tractor. A power transmission system consisting of electrical and mechanical components for each axle is shown in Figure 1. Each motor operated under harsh conditions while receiving continuous power during operation. We installed additional mechanical systems, including gear reducers, wheels, and an electrical system with a generator and battery packs. In total, four electric motors were installed, one for each wheel, to improve the output and traction performance of the tractor.

### 2.2. Configuration of the Electric AWD Tractor

**Figure 2:**
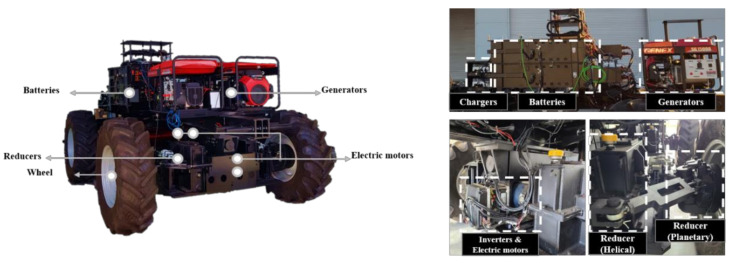
Configuration of the electric AWD tractor (reproduced from [29]).

The electric AWD tractor uses a platform developed in previous research [29]. Figure 2 shows the configuration of the electric AWD tractor with dimensions of 5500 × 2500 × 1950 mm^3^ (length × width × height); the empty vehicle mass was 7440 kg. The weight distribution ratio of the front and rear was designed to be as similar as possible when developing the platform. Table 1 lists the components and specifications of the electric AWD tractor. The battery type was LiFePO_4_, which has a long cycle and can adequately cope with the high power output of the electric motor during operation [30]. The total battery capacity was 58.4 kWh, and the rated voltage and discharge rate were 70.4 V and 2 C (30 min. discharge), respectively. High battery capacity is suitable for agricultural operations that require high torque; therefore, the use of four batteries increased the usable time since they powered only one motor each. The C-rate was selected in consideration of battery damage protection, while the required current of the electric motor dramatically increased due to high load. The tractor was equipped with a rear three-point hitch, so the batteries were installed close to the front axle to provide adequate weight on the front tires when the implement was in the raised position. Two 13.5 kW-class generators (GENEX ST15000, Honda, Tokyo, Japan) each powered by a gasoline engine supplied electrical energy to the batteries and the electric motors. The electric AWD tractor performs agricultural work based on the energy stored in the batteries, and the generator is used only when there is an urgent need for energy supply due to battery power shortage or breakdown during agricultural work. Separate from using the generators to charge the batteries, charging was possible with a charger that was compatible with a 220 V outlet or with the current generated by regenerative braking. The maximum output current of the charger was set at 50 A, considering the safety of battery charging. Four electric motors were used for turning radius reduction and slip control. Each motor was able to output appropriate torque, thereby controlling each wheel independently. A brushless AC type electric motor (HPEVS AC-34, Hi Performance Electric Vehicle Systems, Ontario, CA, USA) that included a controller (Curtis 1238-6501, Curtis Instruments, Inc., Mount Kisco, NY, USA) was selected. The maximum torque and rotational speed of the electric motor were 119.7 Nm and 8000 rpm, respectively, and the power and torque were considered to be appropriate for a tractor for the over-100 kW class. The maximum and rated power of the electric motor were approximately 37 and 30 kW, respectively. Two gear reducers were installed on each motor output shaft to increase the wheel drive torque. The planetary gear reducers used a conventional knuckle arm to facilitate tire mounting on the tractor, and the gear reduction ratio was 12.05. To attain wheel torque sufficient for typical agricultural work, a second gear reducer, which was a helical gear reducer with a reduction ratio of 4.3, was used between the planetary reducer and the motor. We used BKT AGRIMAX RT 855 380/85R24 R-1W agricultural drive tires (BKT, Mumbai, India) on the tractor. The front:rear static weight distribution was optimized using four wheels of the same size, and the current consumption of the battery was kept constant by generating similar torque in the front and rear wheels.

### 2.3. Load Measurement System

**Figure 3:**
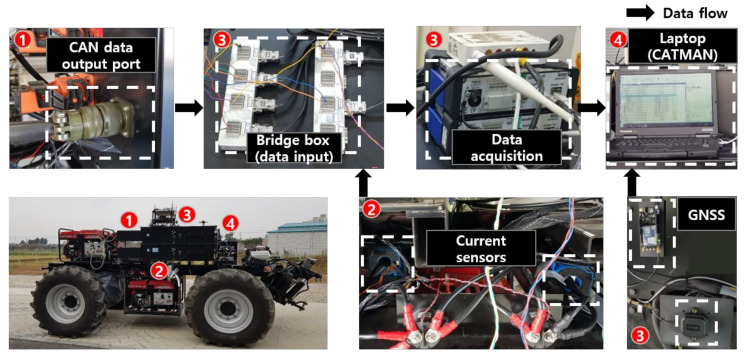
Data measurement system and data flow of the electric AWD tractor.

The data measurement system consisted of current sensors, a controller area network (CAN) logging system, and a data acquisition (DAQ) device, as shown in Figure 3. The motor torque was estimated based on the motor input current, the motor rotational speed, and the battery voltage. The relational equation was obtained using data measured from the dynamometer test of the motor. The motor torque was calculated using the measured data as input values. Therefore, the data measurement system was installed to calculate the motor’s output torque by measuring the output current, rotational speed of the electric motor, and battery voltage. Current data were measured from current sensors (model LF-1005S, LEM USA Inc., Milwaukee, WI, USA), and the rotational speed of the electric motor and battery voltage were measured in real time by configuring the CAN logging system. The current sensors used in this study were installed on the electric wire between each controller of the electric motor and battery. The current sensors generate a magnetic field according to the current flow, and the current generated by the magnetic field is detected using a Hall sensor, up to 1000 mA. The accuracy of the sensor using this method is about ±0.4%, and reliable current data can be obtained. A DAQ (QuantumX MX840B, HBM, Darmstadt, Germany) was installed to acquire the signals from the current sensors, and a CAN logging system was used to transfer them to the laptop computer. The accuracies of the sensors ranged from 0.05% to 0.1%, depending on the sensor technology. The DAQ had eight channels and was capable of 40,000 samples s^−1^ per channel; detailed specifications are shown in Table 2. In the DAQ system, four 24-bit analog input channels with a sampling rate of 200 Hz per channel were used to acquire the current, and two digital input channels with a sampling rate of 200 Hz per channel were used to measure the motor rotational speed and battery voltage that were in the CAN data. The travel speed of the tractor was measured using a global navigation satellite system (GNSS) module (ZED-F9P, u-blox, Thalwil, Switzerland) connected directly to the laptop. The GNSS module used RTK technology, and the horizontal and vertical accuracies were both 0.01 m. The measured data were transmitted and saved on a laptop computer using the signal processing program, CATMAN (ver. 3.1, HBM, Darmstadt, Germany).

### 2.4. Traction Performance Evaluation

**Figure 4:**
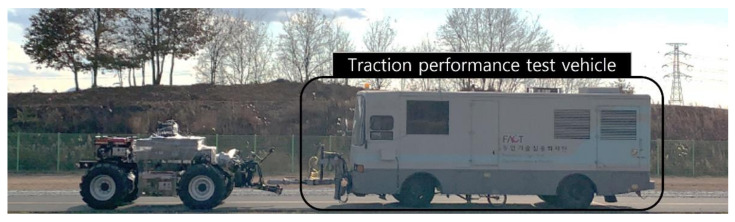
Traction test of the electric AWD tractor using traction performance test vehicle on a concrete surface.

A traction performance test vehicle was connected through a drawbar configured at the rear of the electric AWD tractor to perform the traction test, as shown in Figure 4. The total weight and dimensions of the vehicle were 10,820 kg and 10,915 × 2490 × 3210 mm^3^ (length × width × height), respectively. The traction performance test was conducted on concrete with a slip ratio of 15% or less. The test was conducted at the driving circuit located in Wansan-gu, Jeonju-si, and Jeollabuk-do, Republic of Korea (35°49′41.5″ N 127°03′13.7″ E). The travel speed of the electric AWD tractor was maintained at 4 km/h by driving the electric motor at a constant rotational speed. The electric AWD tractor traveled straight for about 100 s, and the brake system of the traction performance test vehicle was operated irregularly under the condition that the slip ratio did not exceed 15% to measure the traction force under severe conditions.

The axle torque generated the traction of the tractor by the rotation of the drive wheel. The net traction force was calculated from the axle torque during the test, tire diameter, and rolling resistance. Thus, traction performance is closely related to axle load and performance [31]. The traction performance was evaluated by analyzing the relationship with slip ratio as a variable. The slip ratio of each wheel was calculated based on the theoretical speed using the rotational speed of the axle and the actual travel speed measured by the GNSS, and expressed as:

(1) $$s=\left(\frac{V_{0}-V_{a}}{V_{0}}\right)\times 100\left(\%\right)$$

where s is the slip ratio (%), $V_{0}$ is the theoretical speed (km/h), and $V_{a}$ is the actual travel speed (km/h). The net traction force, DR, and TE were analyzed as indicators of the traction performance [24]. The net traction force is calculated using the gross traction and the rolling resistance [32], represented as:

(2) $$NT_{a}=\frac{T_{a}}{r_{t}}-R_{h}$$

where $NT_{a}$ is the net traction force for one axle (kN), $T_{a}$ is the torque of the single motor (kN·m), $r_{t}$ is the effective torque radius of the tire (m), and $R_{h}$ is the rolling resistance (kN).

(3) $$R_{h}=W\left(0.04+\frac{1.2}{C_{n}}\right)$$

where W is the total weight (kN) of the electric AWD tractor, including the tractor and measurement system, and $C_{n}$ is the wheel numeric.

(4) $$C_{n}=\frac{CIbd}{W}$$

where CI is the ASABE cone penetrometer index of concrete condition (kPa), and b and d are the tire width (m) and the tire diameter (m), respectively, of the electric AWD tractor.

The DR is calculated using the traction force and weight [24], represented as:

(5) $$\mathrm{DR}=\frac{NT}{W}$$

where NT is the net traction force of the electric AWD tractor (kN) and W is the total weight (kN) of the electric AWD tractor.

The driving wheel power of the tractor is calculated using the torque and rotational speed of the axles [19,33], summing the power required by the four wheels. This is represented as:

(6) $$P_{a}=\frac{2\pi TN}{60,000}$$

where $P_{a}$ is the axle power (kW), T is axle torque of the electric AWD tractor, and N is the axle rotational speed (rpm).

The drawbar power is calculated by the traction force and travel speed [34], represented as:

(7) $$P_{db}=\frac{NT\times V_{a}}{3.6}$$

where $P_{db}$ is the drawbar power (kW), NT is the net traction force of the electric AWD tractor (kN), and $V_{a}$ is the actual travel speed (km/h).

TE is an index indicating the ratio of the drawbar power calculated by the net traction force and axle power [35], represented as:

(8) $$\mathrm{TE}=\frac{P_{db}}{P_{a}}$$

where TE is the tractive efficiency.

### 2.5. Agricultural Performance Evaluation

The motors of the electric AWD tractors connect to the same mechanical systems; therefore, the output torque of the motors did not vary significantly from axle to axle. However, when skid steering is adopted [20], a difference occurs in the output torque of the motors located on the left and right sides. The torques of the left and right motors were compared, and the relationship between the rotational speed and torque of the motor was analyzed. The agricultural work performance of the electric AWD tractor was confirmed through the axle torque and compared to the workload data of a conventional tractor measured during agricultural work. The workload data was the axle torque measured during plow tillage, which had the highest power requirements of the medium-to-large tractor (LS1404, LS Mtron Ltd., Anyang, Korea) used mainly in Korea. The travel speed of the tractor was 4.09 km/h, based on a questionnaire related to users’ preferred working speeds in Korea. The engine rated power of the conventional tractor is 112 kW, and this model similar to the specifications of an electric AWD tractor, and the detailed specifications of the conventional tractor, implements and conditions of field test were shown in Table 3. The workload data measured in the previous study [36,37,38] was used for analyzing and comparing the axle load and agricultural performance. The axle speed and torque of the electric AWD tractor were indicated by applying the reduction ratio to the electric motor. The applied curve and workload data were indicated on the same graph to determine the workability.

### 2.6. Statistics Analysis

In order to validate the drawbar power, axle power, tractive efficiency, and dynamic ratio for the electric AWD tractor, the linear regression analysis were performed. The linear regression analysis was performed using statistical software, IBM SPSS Statistics (SPSS 24, IBM Corp., Armonk, NY, USA). The correlation of the data between slip and each factor was compared and analyzed using *R*-squared (*R*^2^). *R*^2^ is a statistical measure that represents the proportion of the variance for a dependent variable, and it is explained by an independent variable or variables in a regression model. The *R*^2^ through regression analysis was calculated through Equation (9).

(9) $$R^{2}=\frac{\sum_{i}{\left({\hat{y}}_{i}-\bar{y}\right)}^{2}}{\sum_{i}{\left(y_{i}-\bar{y}\right)}^{2}}=1-\frac{\sum_{i}{\left(y_{i}-{\hat{y}}_{i}\right)}^{2}}{\sum_{i}{\left(y_{i}-\bar{y}\right)}^{2}}$$

where *R*^2^ is the coefficient of determination of the regression equation, $y_{i}$ is the y value for observation i, $\bar{y}$ is the mean of y values, and ${\hat{y}}_{i}$ is the predicted value of y for observation i.

## 3. Results

### 3.1. Drive Performance of the Electric AWD Tractor

#### 3.1.1. Performance of Electric Motors

**Figure 5:**
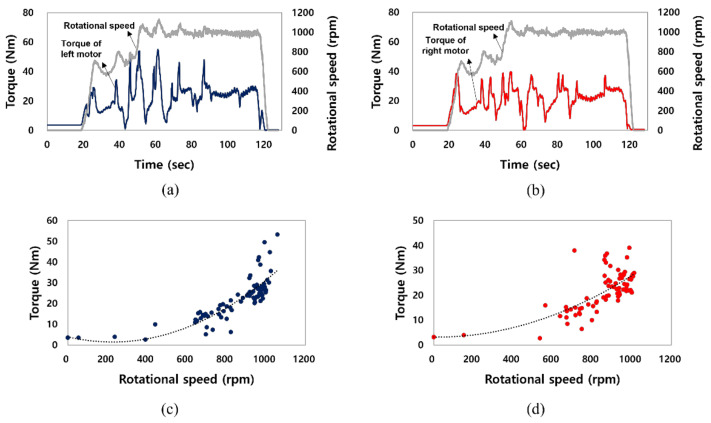
Results of the electric motor torque and rotational speed tests: (**a**) torque and rotational speed of the left (front) motor as time, (**b**) torque and rotational speed of the right (rear) motor as time, (**c**) torque of the left (front) motor as rotational speed, and (**d**) torque of the right (rear) motor as rotational speed.

Figure 5a,b show the output torque and rotational speed of the electric motor mounted on the left (front) and right (rear) wheels of the electric AWD tractor. The rotational speed of the electric motor was maintained in the range of 1000 to 1200 rpm from 30 s after starting through the acceleration section. Figure 5c,d show the relationship between the torque and rotational speed of the electric motor (left and right) during the traction test. The electric motor torque showed a tendency to increase proportionally as the rotational speed increased. The torque also increased in the section where the rotational speed of the electric motor rapidly increased. The output torque of the electric motors were in the range of approximately 0 to 53 and 0 to 39 Nm on the left and right sides, respectively. The torque of the left motor was about 14 Nm higher than the right when skid steering was performed to make the tractor drive straight. The torque of the motor tended to increase as the rotational speed increased in both cases. The torque that appears in the negative direction of the y-axis was generated by the reverse rotation of the motor, as the load increased while the brake system of the traction performance test vehicle was operated.

#### 3.1.2. Axle Torque

**Figure 6:**
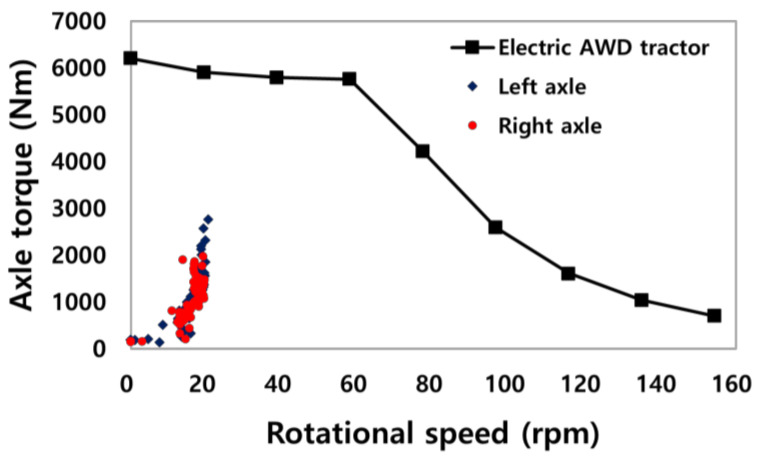
Comparison of the left and right axles of the electric AWD tractor and the T–N curve of the electric motor with gear reduction ratio.

Figure 6 shows the comparison of the torque of left and right axles (applied gear reduction ratio to the measured motor torque) and the torque and rpm (T–N) curve of the axle (applied gear reduction ratio). The axle torque of the electric tractor was calculated by applying a reduction ratio of 51.8 to the torque of the electric motor. The axle torque is increased by 51.8 times compared to the electric motor, and the axle rotational speed is reduced by 51.8 times compared to the electric motor. The maximum torque of axle is approximately 6216 Nm, and the maximum travel speed of the tractor is 36.1 km/h, as each wheel rotates at 160 rpm under the condition of the maximum rotational speed of the motor. The tractor’s traction operation was performed at a travel speed of 4 km/h with a wheel rotational speed of 20 rpm. The torque of the axle on the left (front) side and right (rear) side were in the ranges below 3000 Nm and 2000 Nm, respectively, during the traction test in concrete condition. However, considering the ratio of the load occurring under concrete and soil conditions [29], it was determined that the traction operation could be performed with a high load during agricultural work.

**Figure 7:**
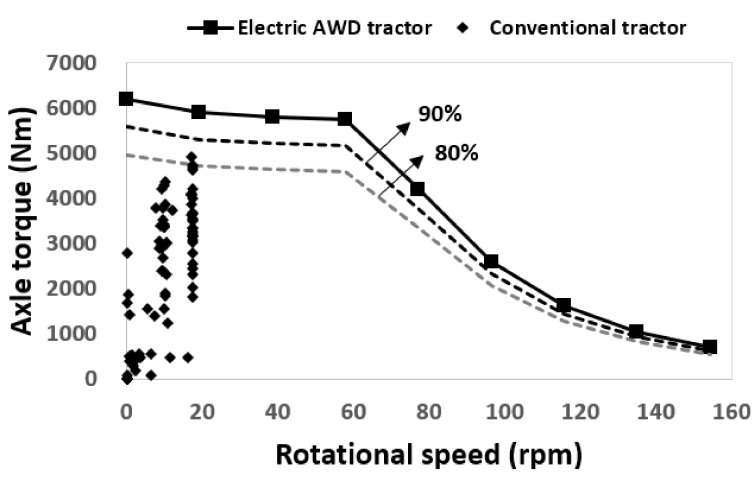
Analysis of agricultural work performance using workload of the conventional tractor and T–N curve of the electric motor with gear reduction ratio.

The agricultural work performance was evaluated using the axle torque of the conventional tractor during plowing tillage, which generated the largest load during operation, and it was compared with the T–N curve of the electric motor applying a reduction ratio, as shown in Figure 7. The maximum value of the axle torque of the conventional tractor was 4912 Nm, which was found in more than 80% of the electric motor output torque ranges. The electric motor could manage the load generated during agricultural work, but the axle torque may increase depending on the weight, soil conditions, and working depth. For stable torque output, using a motor controller with a high output current or increasing the gear reduction ratio is possible to perform even more intense operations.

### 3.2. Traction Performance of the Electric AWD Tractor

#### 3.2.1. Drawbar Power

**Figure 8:**
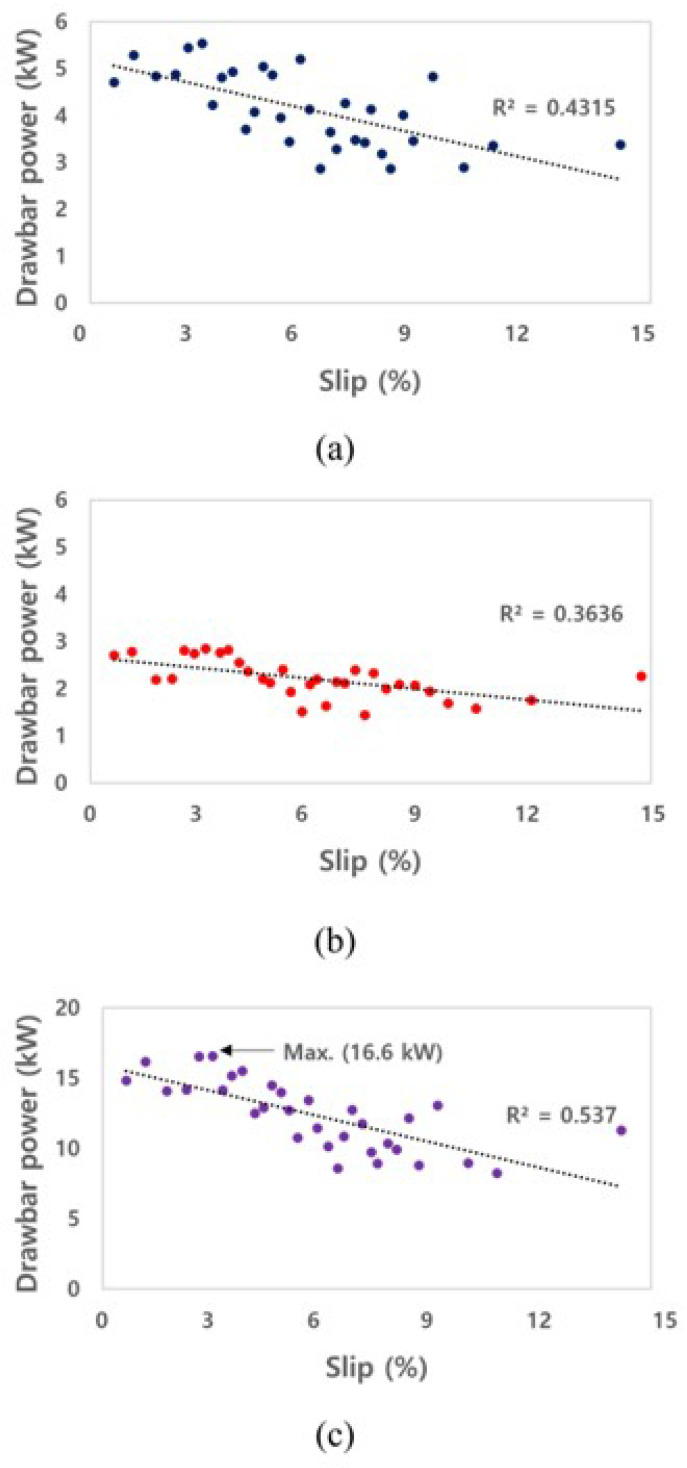
Results of drawbar power as slip for the electric AWD tractor during traction test: (**a**) a left axle, (**b**) a right axle, and (**c**) sum of all axles.

Figure 8 shows the drawbar power on a left axle, a right axle, and the sum of all axles of the electric AWD tractor during traction operation. Drawbar power was calculated using net traction force and travel speed data measured by a GNSS. The maximum value of the drawbar power was approximately 5.5 and 2.9 kW on the left and right axles, respectively, and the maximum drawbar power of the electric AWD tractor was calculated at approximately 16.6 kW during the traction test. Since the weight distribution ratio of front and rear are the same, it has been confirmed that the front and rear outputs are the same, but there is a difference in the output generated from one side (left or right). It is judged that the skid steering greatly influenced this difference. Therefore, the output data for the left and right axle were sufficient to explain the characteristics of the electric AWD tractor. The drawbar power was measured under the conditions that the maximum load was not generated, and the maximum torque of the electric motor was not used. Thus, the traction output will be higher when the work is performed under harsher conditions, such as a soil environment. The drawbar power decreases because the electric motor reduces output power by the controller to protect the system as the slip increases.

#### 3.2.2. Axle Power

**Figure 9:**
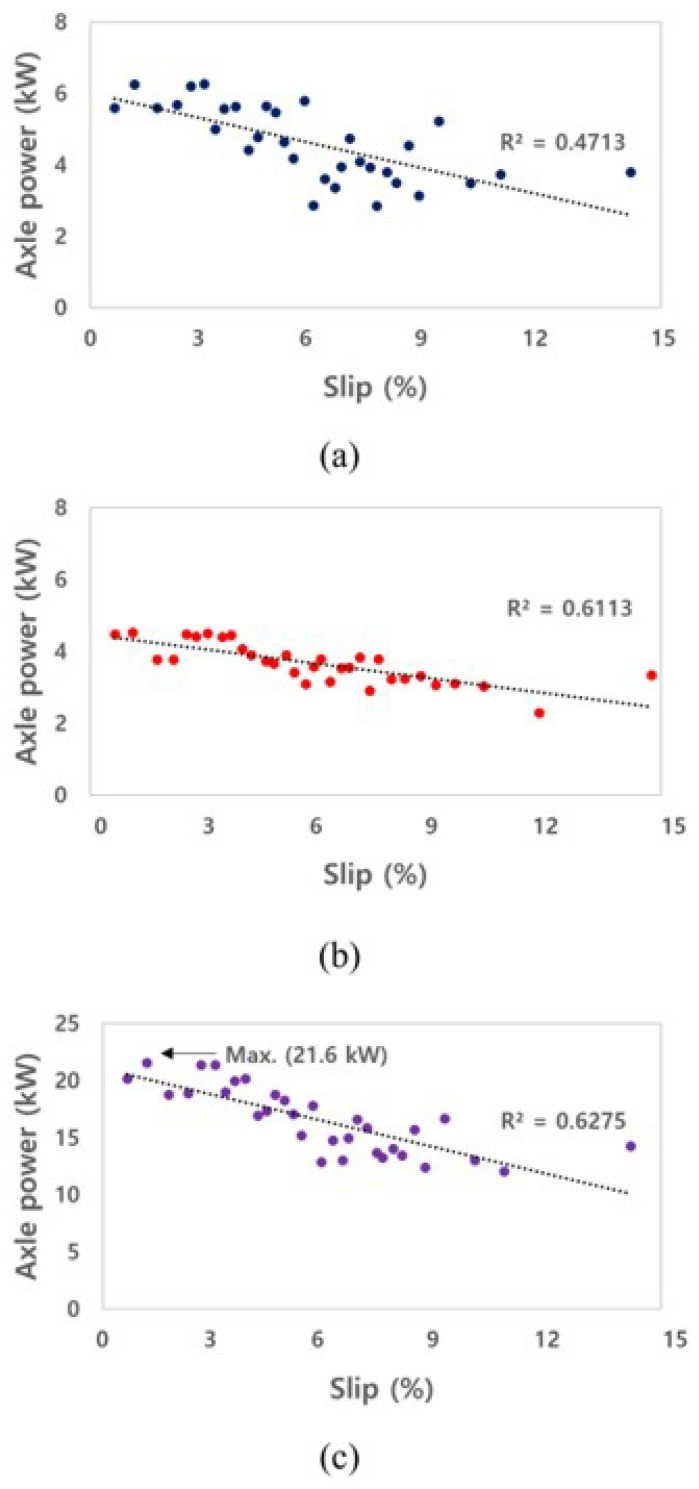
Results of axle power as slip for the electric AWD tractor during traction test: (**a**) a left axle, (**b**) a right axle, and (**c**) sum of all axles.

Figure 9 shows the axle powers on a left axle, a right axle, and sum of all axles of the electric AWD tractor that were measured during the traction test. The axle power was calculated using the torque and rotational speed of the electric motor and the gear ratio of the reducer. The maximum axle power was approximately 6.3 and 4.5 kW at the left and right of the front axle, respectively, and the maximum axle power of the electric AWD tractor was calculated as approximately 21.6 kW in the traction test. The axle power tended to decrease as the slip increased, limiting the output from the motor controller. In particular, the torque and power of the electric motor decreased when the slip increased, as the rotational speed was constantly controlled due to the load generated in the traction performance test vehicle.

#### 3.2.3. Tractive Efficiency and Dynamic Ratio

**Figure 10:**
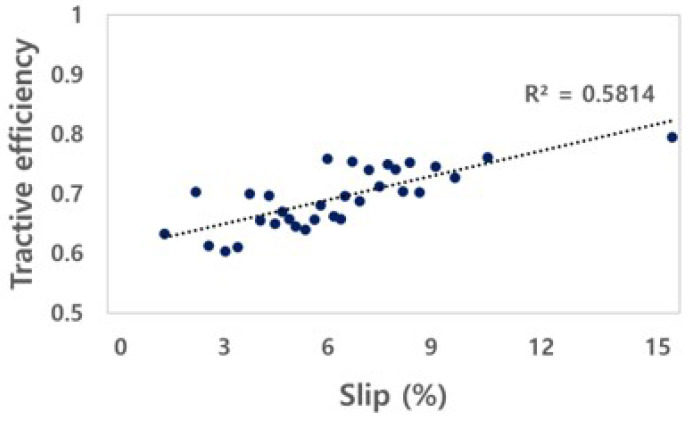
Results of tractive efficiency as slip for the electric AWD tractor during traction test.

The TE of the electric AWD tractor was calculated through the drawbar power and axle power, as shown in Figure 10. The TE of the electric AWD tractor is in the range of 0.60 to 0.80, and the *R*^2^ was found to be 0.58. The increase in tractive efficiency due to slip indicates that the axle power is converted into drawbar power in harsh conditions with a small loss. The tractive efficiency of electric AWD tractors can be increased by improving the motor output.

**Figure 11:**
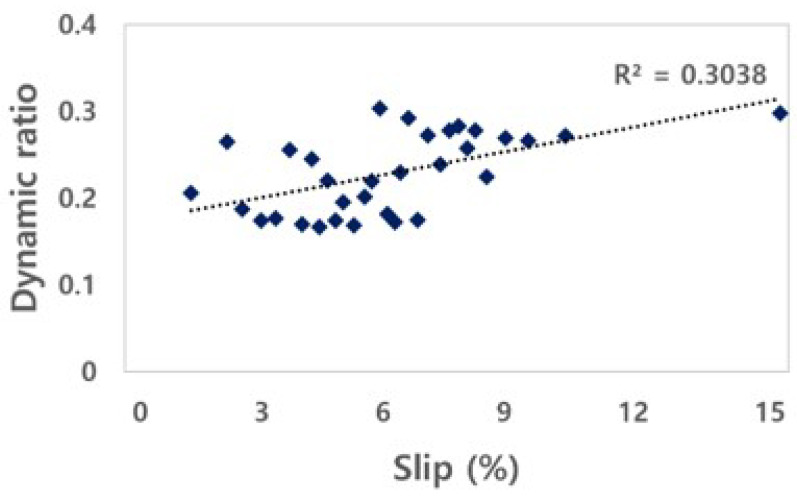
Results of dynamic ratio as slip for the electric AWD tractor during traction test.

The DR is the ratio of traction to the weight acting on the axle. The DR of the electric AWD tractor was calculated from the net traction force and the load applied to the left and right axles, as shown in Figure 11. The ranges of the DR of the electric AWD tractor was in the range of 0.17 to 0.30. The electric AWD tractor had a weight similar to that of a conventional tractor and a lower DR compared to that of conventional tractors because it outputs a relatively low torque due to the motor’s protective control system. The traction factor can be increased to a level similar to that of conventional tractors by increasing the motor output by reducing the weight of the tractor and improving the motor’s self-protection algorithm. The self-protection algorithm is related to the stalling of the motor that limits the output of the motor in order to prevent a malfunction caused by the momentary occurrence of a high load on the motor. This characteristic should be improved by modifying the output control algorithm of the inverter, to perform agricultural work at a higher output, even when a load occurs.

## 4. Discussion

**Figure 12:**
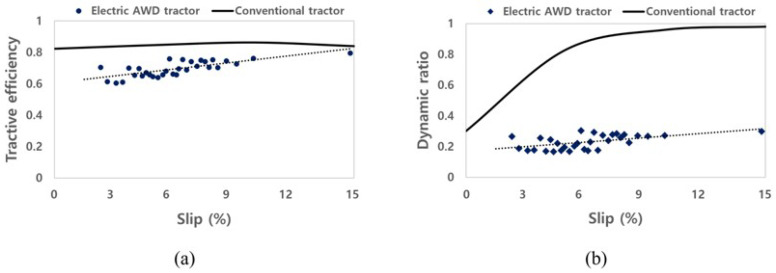
Analysis of traction performance as slip for the electric AWD tractor: (**a**) tractive efficiency, and (**b**) dynamic ratio.

In this study, the traction performance of the electric AWD tractor was evaluated using a traction performance test vehicle. The torque of the electric motor generated during the traction test was found to be within the possible output range of the electric motor through comparison with the performance curve. Since this study was conducted on concrete, the workload of a conventional tractor was used to evaluate the potential to performing agricultural operations. In addition, the traction performance on concrete of the electric AWD tractor should be compared and analyzed by referring to previous traction test studies (on concrete) for conventional tractors. The TE of the conventional tractor in the concrete condition refers to the data of previous studies [39,40,41]. Bashford et al. (1985) presented the tractive efficiency of a four-wheel drive tractor considering weight distribution at a travel speed of 4 km/h in concrete conditions, and the TE was found to be about 0.85 on average. Leviticus and Reyes (1985) classified the TE according to the slip of the tractor in concrete conditions using the concept of a loading factor. The loading factor is determined by the drawbar power, and it is divided into six stages from 0 to 90 kW. The drawbar power of the electric AWD tractor during the traction test is about 16 kW, which corresponds to the second stage (15 to 30 kW). Zoz (1972) presented the TE of the tractor in concrete conditions according to the tire, and the average TE was 0.77. The TE of the tractor in the concrete condition was similar in all studies, and in this study, the average value of the TE of each study was used to consider the various conditions. The comparison results of TE of the conventional tractor referring to previous studies and the electric AWD tractor calculated in this study are shown in Figure 12a. For the TE of the conventional tractor, data within the range of 0 to 15% were used, and the TE ranged from 0.82 to 0.86. The average TE during the traction test of the conventional and electric AWD tractors was approximately 0.84 and 0.70, respectively. The TE of the electric AWD tractor was approximately 0.14 lower, on average, than for the conventional tractor. The DR of the conventional tractor utilized the data of a four-wheel-drive tractor equipped with a radial ply tire, and additionally considered a loading factor [40]. The DR of the conventional tractor in the range of 0 to 15% was used, and ranged from 0.30 to 0.98. The average DR of the conventional and electric AWD tractors was 0.77 and 0.22, respectively, as shown in Figure 12b, and the tendency and difference were high, especially in the high slip section. As a result, the TE of the electric AWD tractor was found to be similar to the results of the previous study. However, the DR of the electric tractor was shown to relatively constant, and it was not similar to that in the results of previous research in the high slip section owing to the characteristics of the electric motor. In general, by increasing the output of the vehicle’s engine/motor, securing sufficient grip on the contact wheel can increase the tractive efficiency. However, the motor used in this study could not secure sufficient output due to stalling. Therefore, to secure sufficient output and improve traction efficiency, it is necessary to consider replacing an inverter capable of high output. It is very important to verify the output performance and detailed control method of the electric motor according to the workload, and to secure the performance that can replace the conventional tractor by improving the limited characteristics of the motor. The improvement of the protection algorithm and the level of agricultural workload should also be considered when designing a tractor with an electric motor and a mechanical system. The results also can be used for research related to the development of a hybrid tractor and battery electric tractor in the process of selecting parts such as motors, batteries, and reducers. In addition, it suggests a direction for the improvement of various electric drive platforms that are applicable to the AWD system.

## 5. Conclusions

This study aimed to design, develop, and evaluate the traction performance of an electric AWD tractor based on the power transmission and electric systems. A load measurement system was employed to collect the data for traction performance evaluation during the traction test. The traction was calculated using torque that was measured using the load measurement system. Traction performance was evaluated using two metrics—TE and DR—of the electric AWD tractor. The TE was calculated using drawbar power and axle power, and the DR was calculated using net traction force and the weight of the tractor. The results indicate that the proposed electric AWD tractor demonstrates improved traction performance under high-load conditions, based on the output torque of the electric motor, TE, and DR. The TE of the electric AWD tractor was in the range of 0.60 to 0.80. The DR of the electric AWD tractor was in the range of 0.17 to 0.30. The traction performance of the electric AWD tractor was compared to that of a conventional tractor, as per the results of a previous study, and the range of slip was 0 to 15% considering the motor and conditions of the traction test. The average TE of the conventional tractor and electric AWD tractor were approximately 0.84 and 0.70, respectively, and the difference was around 0.14. The average DR of the conventional and electric AWD tractors were 0.77 and 0.22, respectively, and the tendency and difference were high. This result was due to the motor’s self-protection algorithm. A low DR means that the output power per weight is insufficient; therefore, the power-limiting algorithm of the electric motor must be improved according to slip. In a future study, an improved motor control algorithm will be applied to allow the performance of actual agricultural operations, and the traction performance of AWD tractors in various conditions (i.e., soil environment, with different implements, etc.) will be evaluated.

## Figures and Tables

**Table 1:** Components and specifications of the electric AWD tractor.

Item		Specifications
Length × Width × Height (mm^3^)		5500 × 2500 × 1950
Mass (kg)		7440
Electric motor	Max. torque (Nm)	119.7
	Max. rotational speed (rpm)	8000
	Max. power (kW)	37
Battery	Capacity (kWh)	58.4
	Type	LiFePO_4_
	Voltage (V)/C-Rate (C)	70.4/2
Reducer	Planetary gear ratio	12.05
	Helical gear ratio	4.3
Tire		380/85R24
Generator	Rated power (kW)	13.5
Charger	Max. output current (A)	50

**Table 2:** Specifications of DAQ used in this study.

Item	Specifications
Company	HBM
Model	QuantumX MX840B
Length × Width × Height (mm^3^)	52.5 × 200 × 121
Accuracy class (%)	0.05–0.1
Operating temperature (°C)	−20–65
Number of channels	8
Max. sample rate per channel (kS·s^−1^)	40

**Table 3:** Specifications of the conventional tractor, implements, and conditions of field test.

Item		Specifications
Tractor	Company	LS Mtron Ltd.
	Model	LS1404
	Length × Width × Height (mm^3^)	4482 × 2320 × 3098
	Mass (kg)	6080
	Rated power of engine (kW)	112.3 @ 1799 rpm
Moldboard plow	Company	Woongjin
	Model	WJR4PS
	Length × Width × Height (mm^3^)	3410 × 2120 × 1530
	Mass (kg)	930
Operating condition	Travel speed (km/h)	4.09
	Working depth (cm)	17–20
Soil environment	Cone index (kPa)	1115
	Water content (% dry basis)	24.6
